# Effects of Scapulothoracic Physical Therapy on Shoulder Function in Adhesive Capsulitis: A Systematic Review and Meta-Analysis

**DOI:** 10.7759/cureus.113139

**Published:** 2026-07-22

**Authors:** Roopa Rao, Suraj Kanase

**Affiliations:** 1 Department of Physiotherapy, Krishna College of Physiotherapy, Krishna Vishwa Vidyapeeth (Deemed To Be University), Karad, IND

**Keywords:** adhesive capsulitis, physical therapy, rehabilitation, scapulothoracic joint, systematic review

## Abstract

Adhesive capsulitis (AC), also known as frozen shoulder (FS), is a common debilitating condition characterized by progressive pain, severe restriction of the shoulder range of motion (ROM), and functional disability. While conventional physical therapy remains the cornerstone of conservative management, the additional benefit of interventions specifically targeting the scapulothoracic joint has not been systematically evaluated. We conducted this systematic review and meta-analysis to determine whether physical therapy targeting the scapulothoracic joint provides a measurable additive benefit over conventional therapy in patients with AC, with emphasis on pain, shoulder ROM, disability, and scapular kinematics. We systematically searched PubMed, Scopus, CINAHL, EBSCO, ProQuest, and Cochrane CENTRAL databases without any year restrictions. Following a thorough, systematic screening by two independent reviewers, nine randomized controlled trials (RCTs) involving 466 participants met our inclusion criteria. A random-effects model was used to conduct meta-analysis. The GRADE framework was used to assess the certainty of evidence. The pooled analysis showed no statistically significant between-group difference in pain (SMD = -0.28; 95% CI -1.11 to 0.54). However, scapulothoracic interventions significantly improved shoulder flexion ROM (SMD = 0.57; 95% CI 0.16 to 0.98; p < 0.05) and functional disability as measured by the Shoulder Pain and Disability Index (SPADI) (SMD = −0.87; 95% CI -1.55 to -0.19; p = 0.012), with a GRADE certainty rated high for the SPADI finding. Effects on abduction and external rotation ROM were positive but could not reach statistical significance. Sensitivity analyses restricted to low-risk-of-bias and studies with some concerns confirmed the robustness of the flexion result and revealed a significant abduction effect (SMD = 0.68; p = 0.029) that was obscured in the primary analysis. These findings support the inclusion of scapulothoracic interventions into physical therapy programs for AC. Future high-quality RCTs with larger samples and standardized protocols are needed to consolidate these results.

## Introduction and background

Adhesive capsulitis (AC), commonly referred to as frozen shoulder (FS), is characterized by a gradual onset of pain and a progressive reduction in both active and passive glenohumeral range of motion (ROM). The condition is typically self-limiting. This idiopathic condition arises from capsular fibrosis with markedly reduced capsular volume [1,2]. It affects approximately 2%-5% of the general population, with a higher incidence seen among individuals aged 40-60 years, women, and those with diabetes mellitus [1,2]. Clinically, AC unfolds across three stages: a painful stage, a progressively stiffening (frozen) stage, and a final thawing stage, in which motion gradually returns [1]. The normal shoulder mechanics, which contribute to the scapulohumeral rhythm (SHR) and efficient load transfer across the upper extremity, are disrupted in AC. Increased scapular external rotation, along with reductions in posterior tilt and upward rotation, has been consistently documented [3], possibly adding to the glenohumeral restriction. Although exercise and physiotherapy modalities are well established in the conservative management of FS, most treatment programs have traditionally focused on glenohumeral pain relief and ROM recovery, with relatively little emphasis on the scapula's contribution to overall shoulder function. Recent evidence from scapular-focused rehabilitation programs, including proprioceptive neuromuscular facilitation (PNF) techniques targeting scapular elevation and depression patterns, suggests that integrating scapular exercises with standard physiotherapy may yield additional benefits for pain reduction, strength gains, and improved functional outcomes [1]. Thus, the need for interventions targeting the scapula can be rationally justified in patients with FS [3]. Although previous systematic reviews have examined scapular-based interventions in broader shoulder conditions, an important gap remains regarding their specific effectiveness in AC. Melo et al. [3] reviewed specific scapular therapeutic exercises in patients with shoulder pain, but their review included a heterogeneous shoulder pain population and was not focused specifically on AC. Similarly, Ferlito et al. [4] evaluated interventions that primarily targeted the scapulothoracic complex in patients with subacromial impingement and FS; however, the evidence was combined across different shoulder pathologies, limiting the ability to draw condition-specific conclusions for AC. Hence, there is a need for a focused systematic review and meta-analysis to synthesize the available evidence specifically on the effectiveness of physiotherapeutic interventions targeting the scapulothoracic joint in AC. This focused evidence synthesis may help clinicians select appropriate scapulohumeral rehabilitation strategies for patients with AC.

## Review

Methodology

Study Design

This systematic review was conducted following the Preferred Reporting Items for Systematic Reviews and Meta-Analyses (PRISMA) guidelines [5]. The review was registered with PROSPERO (registration number: CRD420251126688) prior to its commencement.

Search Strategy

A systematic search of PubMed, Scopus, CINAHL, EBSCO, ProQuest, and Cochrane CENTRAL was conducted through the Krishna University online library and external sources. Grey literature was searched using Google Scholar, and relevant published articles were retrieved for full-text screening. The search strings for each database are provided in Table 1. The initial search strategy included randomized controlled trials (RCTs) with no restrictions on the year of publication, recognizing that earlier trials may remain methodologically relevant. The database search was conducted between October 2025 and December 2025. The reference lists of eligible studies were also manually searched to identify additional records. All retrieved records were imported into Rayyan (Rayyan Systems Inc., Cambridge, MA, USA) to facilitate duplicate removal, title and abstract screening, and subsequent full-text review.

**Table 1:** Search strategy *The broader PubMed strategy was used intentionally to enable us to identify possible interventions that could be included in the comparator group, with scapulothoracic specificity applied at the eligibility screening stage.

Databases	Search string	Date searched
PubMed^*^	(((physical therapy modalities[MeSH Terms]) OR ("physical therapy modalities"[Text Word] OR "physical therapy*"[Text Word] OR "physical modalities"[Text Word] OR "physiotherapy modalities"[Text Word] OR "physiotherapy"[Text Word] OR rehabilitation[Text Word] OR "physical rehabilitation"[Text Word] OR "therapeutic modalities"[Text Word] OR "physical therapy techniques"[Text Word])) AND ((bursitis[MeSH Terms]) OR (bursitis[Text Word] OR "inflammation of bursa"[Text Word] OR "adhesive capsulitis"[Text Word] OR "frozen shoulder"[Text Word] OR "shoulder adhesive capsulitis"[Text Word]))) AND (((((comparative effectiveness research[MeSH Terms]) OR (treatment outcome[MeSH Terms])) OR (effectiveness[Text Word] OR efficacy[Text Word] OR effect[Text Word] OR outcome[Text Word])) OR (impact[Text Word])) OR (performance[Text Word]))	Oct-Dec 2025. Coverage: 1946-present. Final search: Oct 2, 2025
CINHAL	MH "Physical Therapy Modalities+" OR MH "Exercise Therapy+" OR TI(Physiotherapy OR "Physical Therapy" OR "Therapeutic Exercise" OR "Exercise Therapy" OR Rehabilitation OR "Manual Therapy" OR Mobilization OR Manipulation OR "Stretching Exercises" OR Kinesiotherapy OR "Physiotherapeutic Intervention*" OR "Physical Therapy Treatment*" OR "Exercise Intervention*") OR AB(Physiotherapy OR "Physical Therapy" OR "Therapeutic Exercise" OR "Exercise Therapy" OR Rehabilitation OR "Manual Therapy" OR Mobilization OR Manipulation OR "Stretching Exercises" OR Kinesiotherapy OR "Physiotherapeutic Intervention*" OR "Physical Therapy Treatment*" OR "Exercise Intervention*")) AND (MH "Scapula+" OR MH "Shoulder Girdle+" OR TI("Scapulothoracic Joint" OR "Scapulothoracic Articulation" OR "Scapulothoracic Motion" OR "Scapular Dyskinesis" OR "Scapular Kinematics" OR "Scapulothoracic Rhythm" OR "Scapular Movement" OR "Scapular Biomechanics" OR "Shoulder Girdle" OR "Scapular Stabilization") OR AB("Scapulothoracic Articulation" OR "Scapulothoracic Motion" OR "Scapular Dyskinesis" OR "Scapular Kinematics" OR "Scapulothoracic Rhythm" OR "Scapular Movement" OR "Scapular Biomechanics" OR "Shoulder Girdle" OR "Scapular Stabilization")) AND (MH "Adhesive Capsulitis+" OR TI("Frozen Shoulder" OR "Shoulder Capsulitis" OR "Periarthritis of Shoulder" OR "Shoulder Stiffness" OR "Shoulder Joint Disorder*" OR "Shoulder Pain") OR AB("Frozen Shoulder" OR "Shoulder Capsulitis" OR "Periarthritis of Shoulder" OR "Shoulder Stiffness" OR "Shoulder Joint Disorder*" OR "Shoulder Pain"))	Oct-Dec 2025 Coverage: 1937-present. Final search: Oct 28, 2025
Scopus	(TITLE-ABS-KEY("Physical Therapy" OR Physiotherapy OR "Therapeutic Exercise" OR "Exercise Therapy" OR Rehabilitation OR "Manual Therapy" OR Mobilization OR Manipulation OR "Stretching Exercises" OR Kinesiotherapy OR "Physiotherapeutic Intervention*" OR "Physical Therapy Treatment*" OR "Exercise Intervention*")) AND (TITLE-ABS-KEY("Scapulothoracic Joint" OR "Scapulothoracic Articulation" OR "Scapulothoracic Motion" OR "Scapular Dyskinesis" OR "Scapular Kinematics" OR "Scapulothoracic Rhythm" OR "Scapular Movement" OR "Scapular Biomechanics" OR "Shoulder Girdle" OR "Scapular Stabilization")) AND (TITLE-ABS-KEY("Adhesive Capsulitis" OR "Frozen Shoulder" OR "Shoulder Capsulitis" OR "Periarthritis of Shoulder" OR "Shoulder Stiffness" OR "Shoulder Joint Disorder*" OR "Shoulder Pain"))	Oct-Dec 2025. Coverage: 1960-present. Final search: Dec 2, 2025
ProQuest	ab("physical therapy" OR physiotherapy OR rehabilitation OR "manual therapy" OR mobilization OR "exercise therapy" OR "therapeutic exercise" OR PNF OR "proprioceptive neuromuscular facilitation" OR "scapular mobilization" OR "scapular stabilization") AND ab(scapulothoracic OR scapular OR scapula OR "shoulder girdle" OR "scapulohumeral rhythm" OR "scapular dyskinesis" OR "scapular kinematics") AND ab("adhesive capsulitis" OR "frozen shoulder" OR "shoulder capsulitis" OR periarthritis OR "shoulder stiffness") Limiters: Scholarly journals; Date: 2010–2025	Oct-Dec 2025. Final search: Oct 28, 2025
Cochrane Central	("physical therapy" OR physiotherapy OR rehabilitation OR "manual therapy" OR mobilization OR "exercise therapy" OR "therapeutic exercise" OR "proprioceptive neuromuscular facilitation" OR PNF OR "scapular mobilization" OR "scapular stabilization"):ti,ab,kw AND (scapulothoracic OR scapular OR scapula OR "shoulder girdle" OR "scapulohumeral rhythm" OR "scapular dyskinesis" OR "scapular kinematics"):ti,ab,kw AND ("adhesive capsulitis" OR "frozen shoulder" OR "shoulder capsulitis" OR periarthritis OR "shoulder stiffness"):ti,ab,kw in Trials	Oct-Dec 2025. Final search: Dec 2, 2025

Inclusion Criteria

The search included English-language RCTs enrolling participants above 18 years with idiopathic AC, in which at least one intervention arm included physical therapy specifically targeting the scapulothoracic joint. The study design and data extraction followed the PICO framework. The outcomes (O) were assessed between baseline and six months; for the purpose of the meta-analysis, we focused on immediate to short-term effects (up to six weeks). We examined pain using Visual Analog Scale (VAS) or Numerical Pain Rating scale (NPRS), shoulder ROM across all planes (flexion, abduction, and external and internal rotation, measured by inclinometer or goniometer), functional disability using Shoulder Pain and Disability Index (SPADI) or Disabilities of Arm, Shoulder, and Hand (DASH), and scapular kinematics, including scapulohumeral rhythm (assessed by three-dimensional motion capture, electromagnetic tracking, or inclinometer).

Exclusion Criteria

Studies were excluded if any intervention arm involved surgical or medical procedures, non-physical therapy interventions such as acupuncture or acupressure, if the article was not published in English, or if the full text was unavailable.

Study Selection Process

**Figure 1:**
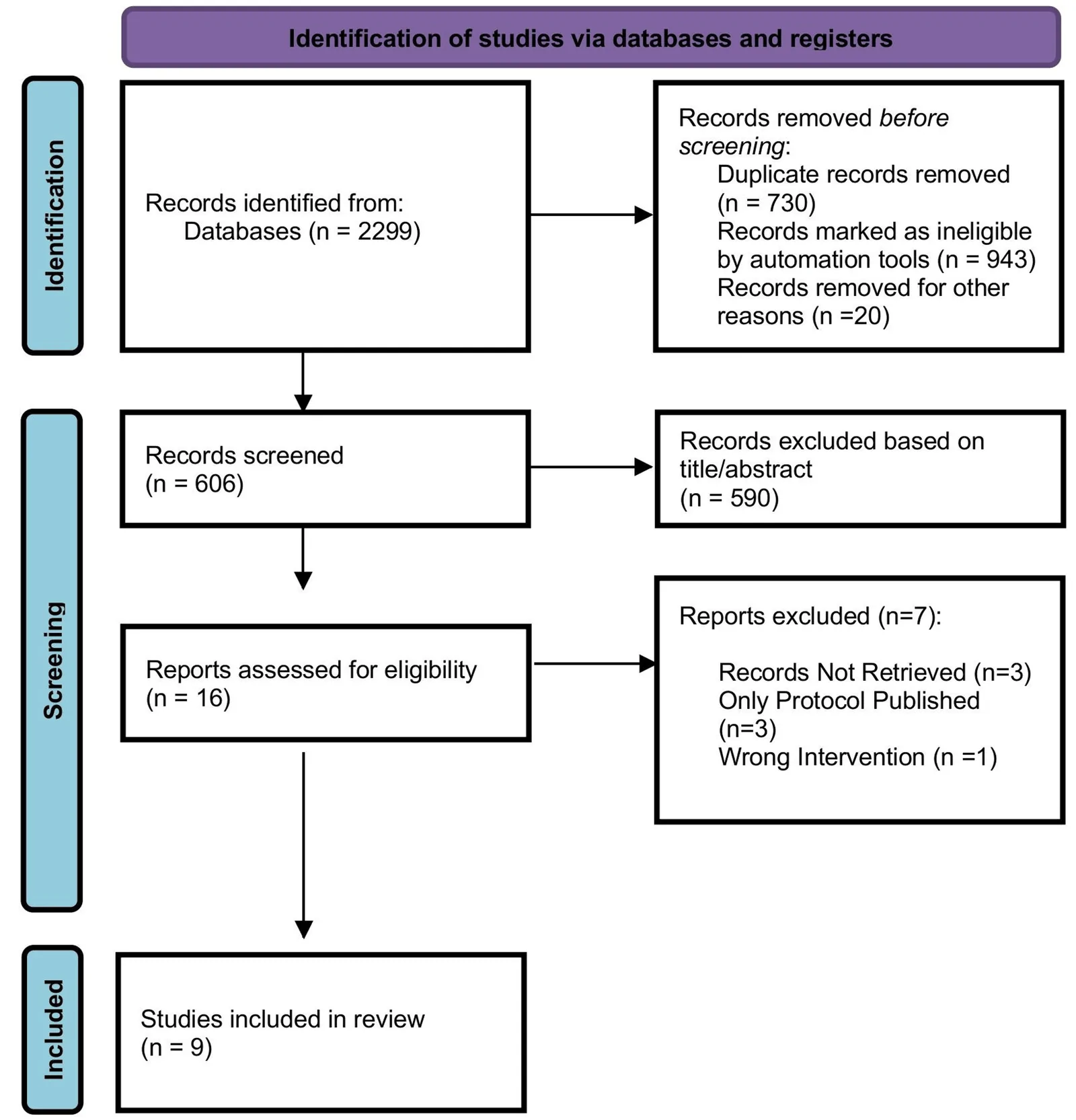
PRISMA flow diagram depicting the article selection process PRISMA: Preferred Reporting Items for Systematic Reviews and Meta-Analysis.

Two primary reviewers (RR and SK) independently screened all records during both the title and abstract screening and the full-text review stages. Disagreements were resolved through discussion; and where consensus could not be reached, a third reviewer (AG) adjudicated. The selection process is documented in the PRISMA 2020 flowchart (Figure 1). 

Data Extraction

Two reviewers (RR and SK) independently extracted data using a standardized form developed a priori by the authors of the review. We extracted the following data: author and year, participant demographics, sample sizes, intervention and comparator descriptions, treatment duration and frequency, outcome measures, follow-up time points, and summary statistics (means, standard deviations, and p-values). Discrepancies were resolved through consensus. When data necessary for meta-analysis were missing or incompletely reported, we contacted the corresponding authors to request the information. If data remained unavailable or were presented in formats that could not be converted to means and standard deviations, such as medians and interquartile ranges, the relevant outcomes were synthesized narratively rather than pooled. Missing statistics were not imputed.

Risk-of-Bias Assessment

**Figure 2:**
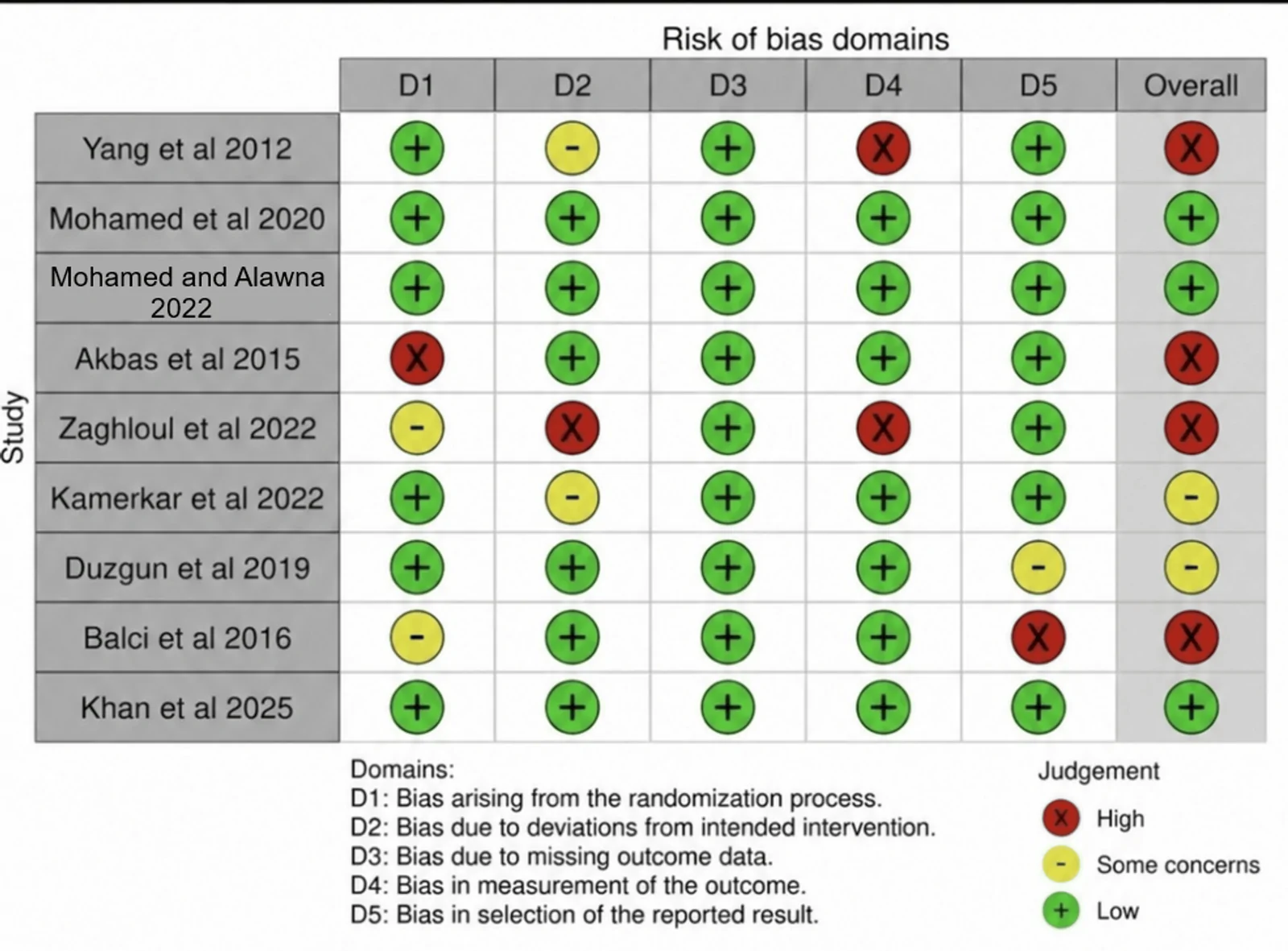
Risk of bias plot of the included studies Source: [1,2,7-13].

**Figure 3:**
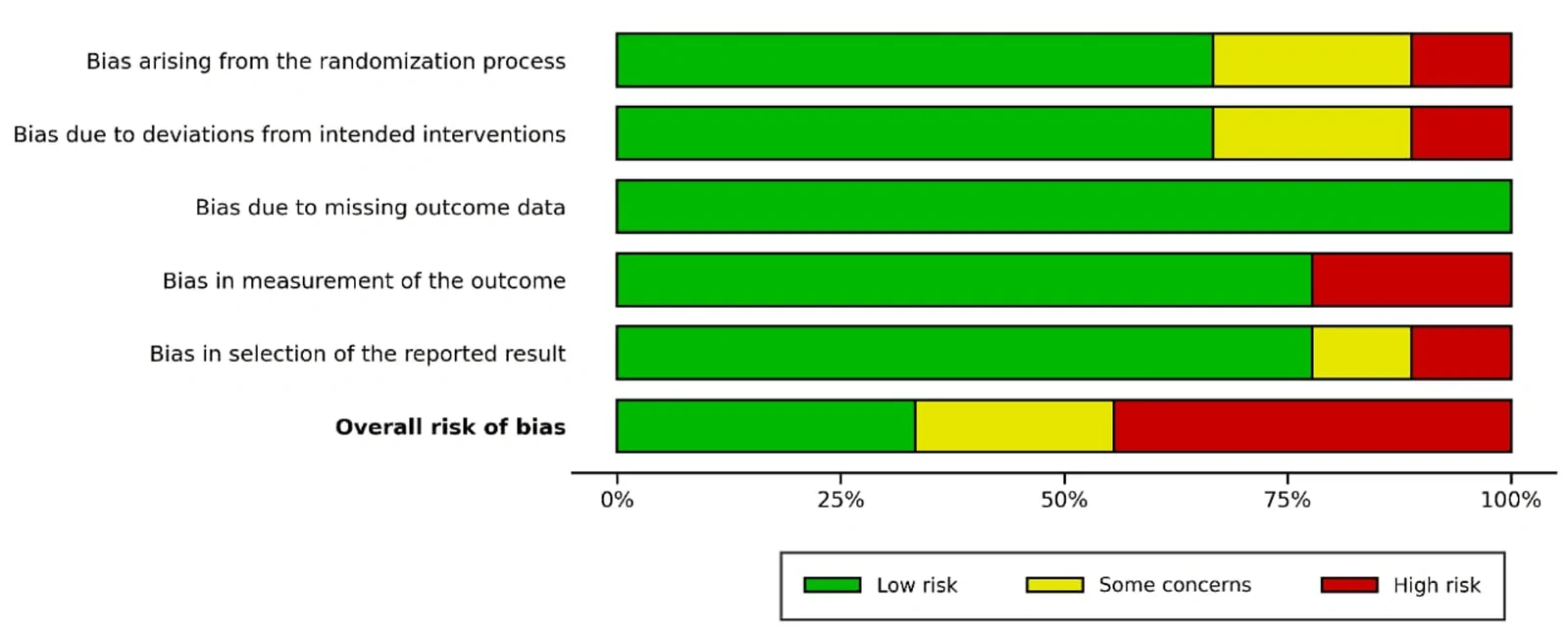
Risk of bias bar chart depicting overall percentage of bias per domain Source: [1,2,7-13].

We assessed the risk of bias [6] using the Cochrane RoB 2 tool, applied independently by two reviewers (RR and SK) across five domains: randomization process, deviations from intended interventions, missing outcome data, outcome measurement, and selection of the reported result. Disagreements were resolved by consensus with a third reviewer (AG). Each study received an overall rating of low risk, some concerns, or high risk. The results are summarized in Figure 2 and Figure 3.

Data Synthesis and Meta-Analysis

A meta-analysis was conducted for three key outcomes: pain, ROM, and disability, for which sufficient comparable data were available. Outcomes exhibiting substantial clinical or methodological heterogeneity were synthesized narratively. Pooled effect sizes were reported as standardized mean differences (SMDs) with 95% CIs, calculated using a random-effects model. Statistical heterogeneity was assessed using the I² statistic and Cochran's Q test (I² < 25% = low; I² 25-75% = moderate; I² > 75% = high). Between-study variance was estimated by τ², and prediction intervals were computed to characterize the expected effect variability across settings. Publication bias was assessed using funnel plots and Egger's test for outcomes with five or more studies. All analyses were performed using NetMetaEasy [14] and independently verified by manual DerSimonian-Laird calculation. To explore the influence of methodological quality on pooled estimates, we conducted pre-specified sensitivity analyses by restricting each meta-analysis to studies rated as having a low risk of bias or some concerns. Primary and sensitivity estimates were compared to assess whether high-risk-of-bias studies materially altered the direction or magnitude of the effects. 

Result

Risk-of-Bias Assessment

Six of the nine studies were rated as having a low risk of bias in the randomization domain. Two studies raised "some concerns." Balcı et al. [1] showed baseline group differences suggesting imperfect randomization, and Zaghloul et al. [7] raised concerns around allocation concealment and was therefore rated as high risk due to concerns about awareness of allocation concealment. One study [8] was judged to be at high risk due to an inadequately concealed randomization sequence. Six studies were rated as having a low risk of deviations from the intended interventions. Kamerkar et al. [9] and Yang et al. [10] raised "some concerns." Kamerkar et al. [9] used an analysis approach not appropriate for their design, while Yang et al. [10] lacked a formal intention-to-treat framework. All nine studies achieved a low risk of bias rating for missing outcome data, indicating adequate follow-up and data completeness across the included trials. Seven studies were rated as having a low risk of outcome measurement. Zaghloul et al. [7] and Yang et al. [10] were rated high risk, with concerns about whether the outcome measures used were appropriate. Seven studies were rated as having a low risk of selective reporting. Duzgun et al. [11] raised some concerns because no preregistered analysis plan was available, making it difficult to verify that all pre-specified outcomes were reported. Balcı et al. [1] also raised concerns over the apparent selective reporting of secondary outcomes. Overall, three studies were rated as having a low risk of bias [2,12,13], two raised some concerns [9,11], and four were rated as having a high risk of bias [1,7,8,10]. The substantial proportion of high-risk studies, reflecting common limitations such as inadequate blinding and selective outcome reporting, meaningfully constrains the certainty of the available evidence.

Qualitative Synthesis of All Studies

All nine included studies were RCTs. One study [12] employed a crossover design, and two studies [1,10] included three groups. Of the nine studies, only three were registered in a clinical trial registry. Six studies were not registered with a clinical trials registry [1,8-12]. The intervention categories are given in Table 2, along with the characteristics of the included studies. 

**Table 2:** Characteristics of the included studies PNF: proprioceptive neuromuscular facilitation, DSR: dynamic scapular recognition, MWM: mobilization with movement, ROM: range of motion, VAS: Visual Analog Scale, NPRS: Numeric Pain Rating Scale, SPADI: Shoulder Pain and Disability Index, LSST: lateral scapular slide test, SST: simple shoulder test, SUR: scapular upward rotation, Flex-SF: Flexilevel Scale of Shoulder Function, TENS: transcutaneous electrical nerve stimulation, US: ultrasound.

Study (author, year)	Participants (n, age)	Experimental	Comparator	Dose	Outcome measures and time points	Main findings	Overall risk of bias
Balcı et al. (2016) [1]	n = 53; age: 50-60 years	PNF group (PNF and physiotherapy modalities)	Two comparator groups: the classic exercise group (classic exercise and physiotherapy modalities) and the control group (physiotherapy modalities)	A hot pack was applied for 20 minutes, followed by TENS (100 Hz, 60 μs, 20 minutes) and US (1 MHz, 1.5 W/cm², 3 minutes). This was followed by scapular proprioceptive neuromuscular facilitation (PNF) (rhythmic initiation/repeated contractions) or stretching and strengthening exercises in the treatment groups.	Pain intensity was assessed using the VAS, LSST, ROM, and SST immediately after the intervention (at 1 hour)	The VAS and LSST showed no significant differences between the groups after the intervention. Shoulder ROM showed significant improvements in all groups, except for flexion and abduction. After the intervention, there was no significant difference in SST between the groups	High
Mohamed et al. (2020) [2]	n = 66; age: 50-60 years	Dynamic scapular recognition exercise	Placebo treatment	Participants received three sessions per week for two months. Both groups received hot pack therapy for 20 minutes and scapular mobilization for five minutes. Active ROM exercises, including flexion and abduction, were performed for 20 repetitions per set, with five sets per session.	Outcomes included scapular upward rotation, SPADI, and shoulder ROM. Assessments were conducted at baseline, two weeks, two months, and six months	Significant differences were observed between the study and control groups after two weeks, two months, and six months for SUR and shoulder flexion and abduction ROM. External rotation showed significant differences at two and six months. SPADI scores were significantly different between the groups at all assessment time points	Low
Khan et al. (2025) [12]	n = 80; age: 21-70 years	Proprioceptive neuromuscular facilitation	Conventional physiotherapy, including capsular stretching and accessory joint mobilization	The intervention was administered four times per week for six weeks	Outcomes included pain severity using the NPRS, shoulder ROM (abduction, lateral rotation, and medial rotation), and the SPADI. All outcomes were assessed at baseline and at six weeks	PNF exercises significantly reduced pain at rest, during shoulder abduction, and while combing hair. Significant improvements in functional performance were also observed	Low
Zaghloul et al. (2022) [7]	n = 36; age: 33-54 years	Scapular mobilization	Glenohumeral MWM	The intervention was administered four times per week for six weeks	Outcomes included pain severity using the VAS, shoulder ROM (flexion, extension, internal rotation, and external rotation), and the SPADI	At six weeks of treatment, patients who received MWM experienced less pain than those who received scapular mobilization	High
Kamerkar et al. (2022) [9]	n = 40; age: 40-65 years	Scapular mobilization plus conventional treatment	PNF plus conventional treatment	The intervention was administered three days per week for four weeks	Outcomes included pain severity using the VAS, shoulder ROM (abduction and external rotation), and the SPADI	The scapular mobilization group showed a statistically significant improvement in VAS scores compared with the PNF group. It also demonstrated a statistically significant improvement in shoulder abduction. Baseline SPADI scores differed between the two groups. The scapular mobilization group also demonstrated a statistically significant improvement in SPADI scores compared with the PNF group	Some concerns
Akbaş et al. (2015) [8]	n = 36; age: 20-70 years old	PNF application to the upper extremity and scapula	Conventional therapy, including a hot pack, ultrasound, and wall and wand exercises	The home exercise program included ultrasound for 20 minutes and exercises performed 10 times per hour. The PNF group performed exercises five times per week, for a total of 15 sessions	Outcomes included structural deformities assessed using the LSST, pain severity using the VAS, the SPADI, and shoulder ROM	Resting pain did not decrease; however, pain during activity and night pain were reduced in the PNF group. No change was observed in the number of patients with structural deformities. Shoulder flexion and abduction increased in the PNF group after therapy, although no significant difference in LSST was observed between the groups. The reduction in SPADI pain scores was greater in the PNF group	High
Yang et al. (2012) [10]	n = 34; age: 50-60 years	End-range mobilization combined with scapular mobilization and standard physical therapy	Criteria control group and the control group, both of which received standard physical therapy	The intervention was administered twice a week for three months	Outcomes included shoulder ROM, disability assessed using the Flex-SF, and shoulder kinematics measured using the Fastrak system	Humeral external rotation and the hand-behind-the-back movement were similar in the criteria intervention and control groups compared with the criteria control group at four and eight weeks. Analysis of shoulder kinematics at four and eight weeks demonstrated improvements in scapular upward rotation and scapulohumeral rhythm in the criteria intervention and control groups compared with the criteria control group	High
Mohamed and Alawna (2022) [13]	n = 67; age: 40-60 years	Rigid taping and dynamic scapular recognition exercises	Sham taping plus dynamic scapular recognition exercises	The intervention was administered three sessions per week for two months	Outcomes included scapular upward rotation, shoulder ROM, and the SPADI, assessed at baseline, two weeks, two months, and six months.	Scapular dyskinesis and scapular upward rotation showed improvement at all assessment time points. Significant improvements in shoulder flexion and abduction were observed at all assessment time points, while external rotation showed significant improvement after two and six months. SPADI scores also demonstrated significant improvements at all assessment time points	Low
Duzgun et al. (2019) [11]	n = 54; age: 40-65 years	Scapular mobilization	Posterior capsular stretching	Scapular mobilization was performed for 10 repetitions, with 30-second rest intervals between sets. Posterior capsule stretching was performed for 10 repetitions, with each stretch held for 20 seconds and a 30-second rest between repetitions. The combined group received both interventions	Outcomes included shoulder ROM and pain assessed using the VAS, including resting pain, night pain, and pain during activity	No significant differences in pain were observed among the groups. However, both groups demonstrated improvements in shoulder ROM	Some concerns

When all nine included studies were considered, interventions focusing on the scapula showed generally favorable but inconsistent effects across the outcomes. For pain, eight studies provided pain-related outcomes, but only two reported a statistically significant superiority of scapular interventions over comparator treatments [9,12]. One study reported that mobilization with movement was superior to scapular intervention for pain reduction [7], suggesting that scapular approaches may not be uniformly superior to other manual therapy techniques. ROM was assessed in all nine studies. Six studies reported improvements in shoulder ROM following scapular interventions, most commonly in flexion, abduction, and external rotation. The remaining three studies [1,7,11] did not demonstrate significant between-group differences; notably, two of these evaluated only acute effects [1,11]. Overall, the direction of effect favored scapular interventions for ROM, although the magnitude and statistical significance varied across the studies. Functional outcomes were assessed in seven studies. Five studies used the SPADI [2,8,12,9,13] and demonstrated functional improvements following scapular intervention. While one study used Flex-SF [10] and found significant improvement in scapular intervention group, another study that used the Simple Shoulder Test [1] showed limited functional change.

Scapular kinematics were assessed in five studies using heterogeneous methods, including the lateral scapular slide test (LSST), scapular upward rotation measures, and three-dimensional motion analysis. Only two studies [2,13] demonstrated significant improvement in scapular kinematics. Three studies failed to show significant between-group differences, including two that used LSST [1,8], which may be less sensitive to dynamic changes in scapular movement. Therefore, while scapular interventions may influence scapular mechanics, the evidence remains inconsistent due to variability in measurement tools.

Qualitative Synthesis (Excluding High-Risk-of-Bias Studies)

After excluding studies judged to have a high risk of bias, the evidence became more consistent in favor of scapulothoracic interventions, particularly for ROM, disability, and scapular kinematics. Low-risk-of-bias studies [2,9,11-13] demonstrated significant improvements in shoulder ROM, especially flexion, abduction, and external rotation, following interventions such as scapular mobilization, dynamic scapular recognition exercise, scapular PNF, and scapular correction taping. For pain and disability, low-risk-of-bias studies generally showed improvement, particularly when outcomes were measured using SPADI. Dynamic scapular recognition exercises and vertical correction taping demonstrated significant reductions in shoulder pain and disability, with effects maintained for up to six months in some studies. ROM findings also remained favorable. Duzgun et al. [11] found acute improvements in ROM following scapular mobilization, posterior capsule stretching, and combined treatment, although no clear superiority between techniques was observed. This suggests that scapular mobilization may have immediate mobility benefits, but acute studies may underestimate longer-term comparative effects. For scapular kinematics, low-risk-of-bias studies provided stronger evidence that scapular-focused interventions can improve scapular upward rotation and scapular dyskinesis. However, the evidence remains limited because only a small number of studies directly measured scapular kinematics, and measurement methods differed across trials. Overall, including high-risk-of-bias studies produced more heterogeneous and less certain findings, whereas excluding high-risk studies strengthened the conclusion that scapular interventions are beneficial for ROM and disability, with more cautious support for pain reduction and scapular kinematic improvement.

Meta-Analysis

**Figure 4:**
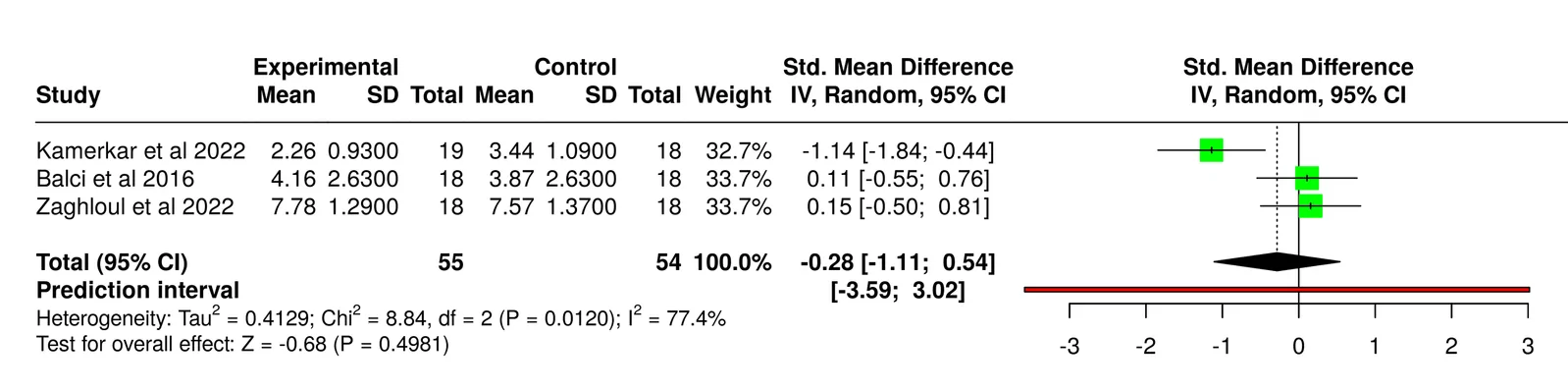
Forest plot for pain assessed using a random-effects model Source: [1,7,9].

**Figure 5:**
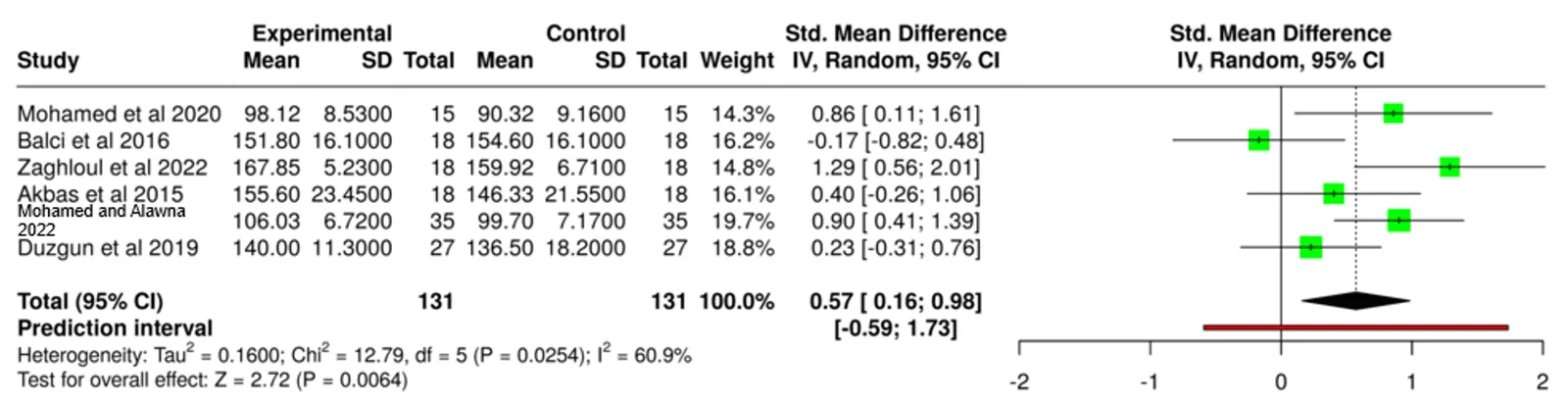
Forest plot for flexion assessed using a random-effects model Source: [1,2,7,8,11,13].

**Figure 6:**
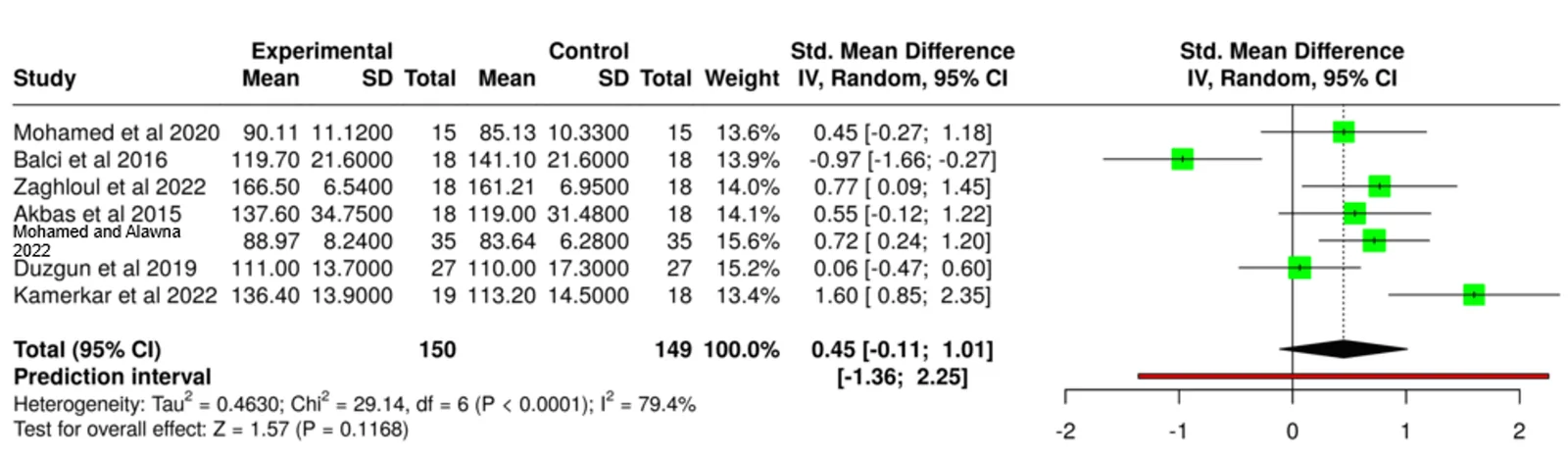
Forest plot for abduction assessed using a random-effects model Source: [1,2,7-9,11,13].

**Figure 7:**
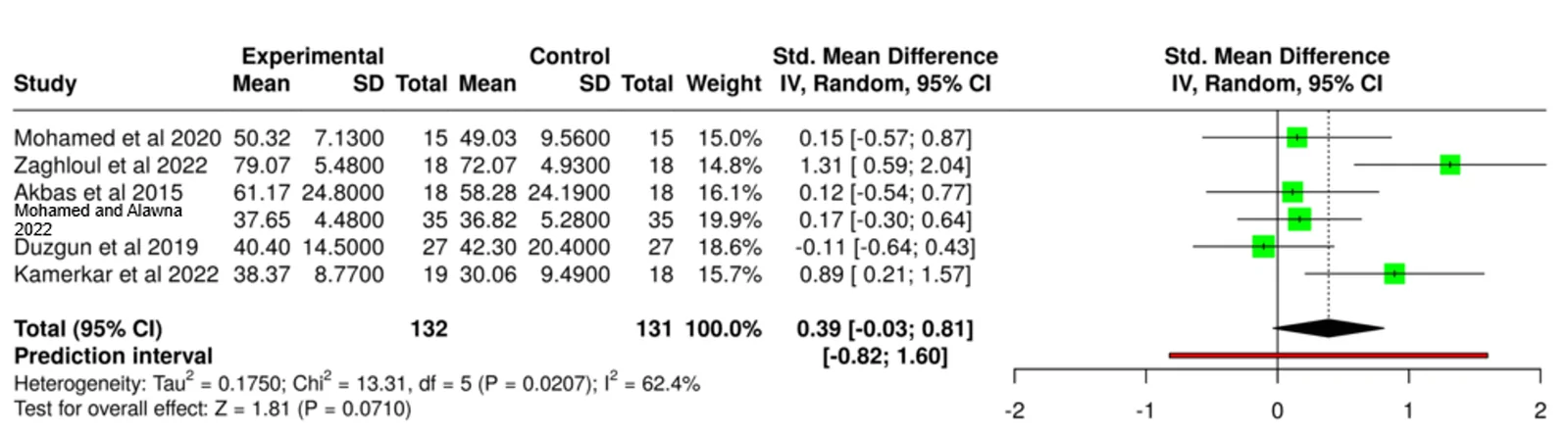
Forest plot for external rotation assessed using a random-effects model Source: [2,7-9,11,13].

**Figure 8:**
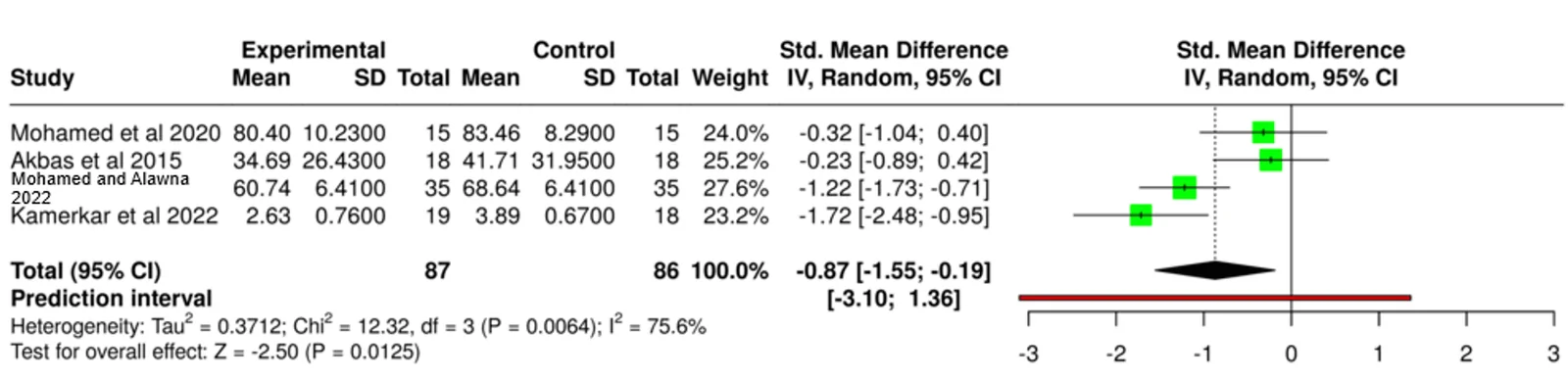
Forest plot for SPADI assessed using a random-effects model Source: [2,8,9,13]. SPADI: Shoulder Pain and Disability Index.

A meta-analysis was performed using NetMetaEasy, a validated, open-access platform for comparative effectiveness research, with a random-effects model and the inverse-variance method [14]. The NetMetaEasy outputs were independently verified by manual DerSimonian-Laird calculation; all core estimates (SMD, 95% CI, I², and τ²) were confirmed. Pooled analyses were conducted for pain (VAS), shoulder ROM (flexion, abduction, and external rotation), and disability (SPADI). Other outcomes demonstrated prohibitive variability in measurement tools and reporting and were synthesized qualitatively. Three studies [1,7,9] were included in the pain meta-analysis (total: 55 experimental and 54 control participants). Considerable heterogeneity was observed across the pain outcome measures. Two studies [2,13] did not assess pain as an independent outcome (the primary outcome was scapular kinematics); one study [8] reported pain as a subdomain of the SPADI; and one study [11] reported median and interquartile range values that could not be incorporated into the pooled analysis. Attempts to contact one author [8] via email for additional data were unsuccessful. The pooled SMD was found to be -0.28 (95% CI -1.11, 0.54), with the prediction interval (-3.59; 3.02) crossing the line of no effect, indicating no statistically significant between-group difference (Z = -0.68, p = 0.498) (Figure 4). Considerable heterogeneity was detected (I² = 77%, τ² = 0.41, p = 0.01), reflecting inconsistent effects across studies in terms of both magnitude and direction. The risk of publication bias was not assessed, as only three studies were included in the analysis. Six studies were analyzed for shoulder flexion ROM (total: 131 experimental and 131 control participants). A pooled SMD of 0.57 (95% CI 0.16-0.98) was obtained, demonstrating a statistically significant small-to-moderate effect favoring scapulothoracic interventions for shoulder flexion ROM (Z = 2.74, p < 0.05) (Figure 5). Moderate-to-high heterogeneity remained (I² = 61%, p = 0.03). No evidence of publication bias was detected (Egger’s test: intercept 0.84, 95% CI -8.26 to 9.94; p = 0.866). Seven studies were analyzed for shoulder abduction ROM (150 experimental and 149 control participants in total). The pooled SMD was 0.45 (95% CI -0.11 to 1.01), with the confidence interval crossing zero, indicating no statistically significant between-group differences (Z = 1.57, p = 0.117) (Figure 6). High heterogeneity was observed (I² = 79%, τ² = 0.46, p < 0.001). The prediction interval (-1.36; 2.25) crossed the null. No evidence of publication bias was detected (Egger’s test: intercept 0.64, 95% CI -10.49 to 11.77, p = 0.915). A total of six studies (132 experimental and 131 control participants) were analyzed for the shoulder external rotation outcome. The pooled SMD was 0.39 (95% CI -0.03 to 0.81), with the confidence interval marginally crossing the line of no effect, indicating no statistically significant between-group differences (Z = 1.83, p = 0.067) (Figure 7). Heterogeneity was calculated to be I² = 62%, with a p = 0.02. No evidence of publication bias was detected (Egger’s test: intercept 5.32, 95% CI -1.60 to 12.24, p = 0.206). Four of nine studies [2,8,9,13] assessed disability using SPADI. The meta-analysis demonstrated a statistically significant moderate-to-large effect favoring scapulothoracic interventions (SMD = -0.87, 95% CI -1.55 to -0.19, Z = -2.50, p = 0.012) (Figure 8). Substantial heterogeneity was observed (I² = 75.6%, τ² = 0.37, p = 0.006). The prediction interval (-3.10; 1.36) crossed the null, indicating variability in the treatment response across study settings. No evidence of publication bias was detected (Egger’s test, p = 0.833).

To assess the robustness of the primary meta-analytic findings, sensitivity analyses were conducted for shoulder flexion, abduction, and external rotation ROM, restricting the pooled estimates to studies rated as low risk of bias or with some concerns using the Cochrane RoB 2 tool. A sensitivity analysis for pain was not performed because only three studies contributed to that outcome. SPADI sensitivity analysis was not indicated, since all contributing studies were assessed as low risk of bias. Restricting the flexion analysis to three low-risk-of-bias studies [2,11,13] yielded a pooled SMD of 0.65 (95% CI 0.19-1.10, p = 0.006), demonstrating that the primary finding remained statistically significant and robust to the exclusion of high-risk-of-bias studies [1,7,8]. Heterogeneity improved markedly (I² = 45.6%, τ² = 0.076), suggesting that methodological limitations in higher-risk studies contributed to the residual variance observed in the primary analysis (I² = 61%). Restricting the abduction analysis to four low-risk-of-bias studies [2,9,11,13] yielded a pooled SMD of 0.68 (95% CI 0.07-1.29, p = 0.029), which achieved statistical significance, in contrast to the primary analysis (SMD = 0.45, 95% CI -0.11 to 1.01, p = 0.117), which did not reach statistical significance. Heterogeneity remained substantial (I² = 72.9%), suggesting true between-study variability among low-risk studies, although the signal was strengthened when higher-risk studies were excluded [1,7,8]. These findings indicate that the nonsignificant primary result for abduction may partly reflect dilution by studies with methodological limitations. Restricting the external rotation analysis to four low-risk-of-bias studies [2,9,11,13] yielded a pooled SMD of 0.24 (95% CI -0.15 to 0.63, p = 0.224), which remained non-significant, consistent with the primary analysis (SMD = 0.39, 95% CI -0.03 to 0.81). Notably, heterogeneity decreased substantially (I² = 43.0% vs 62% in the primary analysis), suggesting that higher-risk studies [7,8] contributed variance without materially altering the direction of the effect. The consistently non-significant results across both analyses reinforce the conclusion that the evidence for scapulothoracic interventions on external rotation ROM remains inconclusive. Collectively, the sensitivity analysis confirmed the robustness of the flexion finding and suggested that the primary abduction result may underestimate the true treatment effect because it included higher-risk studies. The external rotation finding remained unchanged. These results should be interpreted in the context of small sample sizes after study exclusion, which limit the statistical power and generalizability of the sensitivity estimates.

Certainty Assessment

Grading of Recommendations, Assessment, Development, and Evaluation (GRADE) assessment was performed for all outcomes included in the meta-analysis, as shown in Table 3. The certainty of evidence for scapulothoracic interventions compared to conventional physical therapy in the management of AC was assessed across five outcomes. Using the GRADE approach, the quality of evidence was independently assessed by two reviewers according to four levels, ranging from high to very low, consistent with the methodology described by the GRADE Working Group [15]. For pain, substantial heterogeneity was observed (I² = 77%, p = 0.01), indicating variability in both the magnitude and direction of effects across the studies. Downgrading for very serious risk of bias, serious inconsistency, and serious imprecision arising from confidence intervals crossing the minimal threshold of no effect resulted in a certainty of evidence rating of very low. For ROM, moderate-to-high heterogeneity (I² = 61%, p = 0.03) and serious risk of bias in several included studies necessitated downgrading, yielding a certainty rating of very low for the flexion ROM. Abduction ROM, with high heterogeneity (I² = 79%, p < 0.001), resulted in downgrading for serious risk of bias, serious inconsistency, and serious imprecision, yielding a certainty rating of very low. For external rotation ROM with moderate-to-high heterogeneity (I² = 62%, p = 0.02), downgrading for serious risk of bias and serious inconsistency resulted in a certainty rating of very low. The SPADI was statistically significant (p < 0.05); therefore, no downgrading was applied due to concerns about bias, inconsistency, indirectness, or imprecision, and the certainty of evidence was high. Collectively, these findings suggest that scapulothoracic interventions offer a statistically significant advantage for shoulder flexion ROM and disability, as measured by the SPADI, with high-certainty evidence supporting the latter. The effects on pain, abduction, and external rotation were positive but did not reach statistical significance, likely reflecting residual heterogeneity and limited statistical power rather than a true absence of clinical benefit. The very low certainty ratings for pain and most ROM outcomes reflect pervasive methodological limitations, including high risk of bias, substantial statistical heterogeneity, and imprecision of effect estimates. Therefore, clinicians should exercise caution when applying these results to individual patient management while acknowledging the consistently positive direction of the effect across outcomes.

**Table 3:** GRADE assessment RCT: randomized controlled trial, SMD: standardized mean difference, VAS: Visual Analog Scale, ROM: range of motion, SPADI: Shoulder Pain and Disability Index, GRADE: Grading of Recommendations, Assessment, Development, and Evaluations. ^a^The heterogeneity is high. ^b^The confidence interval crosses one threshold. ^c^The confidence interval was not mentioned for a few studies. ^d^The confidence interval crosses two thresholds.

Certainty assessment							Impact	Certainty	Importance
No. of studies	Study design	Risk of bias	Inconsistency	Indirectness	Imprecision	Other considerations			
Pain assessment: VAS [1,7,9]									
3	RCT	Very serious	Serious^a^	Not serious	Serious^b^	None	The experimental cohort included 55 subjects, while the control cohort comprised 54 subjects. The summarized standardized mean difference (SMD) was -0.28, with a 95% CI ranging from -1.11 to 0.54. The overall effect test did not demonstrate statistical significance. Significant heterogeneity was observed (p = 0.01), indicating inconsistency in the magnitude or direction of effects. The I^2^ value of 77% suggests that most of the variability among studies is attributable to heterogeneity rather than random variation	⨁◯◯◯ Very Low^a,b^	Important
Range of motion: flexion (assessed with goniometer/inclinometer) [1,2,7,8,11,13]									
6	RCT	Serious	Serious^a,c^	Not serious	Very serious	None	A total of six studies were analyzed, comprising 131 subjects in both the experimental and control cohorts. The standardized mean difference (SMD) between the two cohorts was 0.57, with a 95% CI of 0.16-0.98, indicating a statistically significant difference. The overall effect was significant (p < 0.05). Significant heterogeneity was observed (p = 0.03), suggesting inconsistency in the magnitude or direction of effects. The I^2^ value demonstrated that 61% of the variability among studies was attributable to heterogeneity rather than random variation	⨁◯◯◯ Very low^a,c^	Important
Range of motion: abduction (assessed with goniometer/inclinometer) [1,2,7-9,11,13]									
7	RCT	Serious	serious^a^	Not serious	Serious^b^	None	Seven studies were included, comprising 150 subjects in the scapular intervention group and 149 subjects in the comparison group. The summarized standardized mean difference (SMD) was 0.45 with a 95% CI of -0.11 to 1.01, indicating no statistically significant difference between the cohorts. The overall effect test did not demonstrate significance. Significant heterogeneity was observed (p < 0.01), indicating inconsistency in the magnitude or direction of effects. The I^2^ statistic showed that 79% of the variability among studies was attributable to heterogeneity rather than random variation	⨁◯◯◯ Very low^a,b^	Important
Range of motion: external rotation (assessed with goniometer/inclinometer) [2,7-9, 11,13]									
6	RCT	Serious	Serious^b^	Not serious	Very serious	None	A total of six studies were analyzed, comprising 132 subjects in the experimental cohort and 131 subjects in the control cohort. No statistical difference was observed between the two cohorts; the summarized standardized mean difference (SMD) was 0.39 with a 95% CI of -0.03 to 0.81. The test for the overall effect did not demonstrate a significant effect. Significant heterogeneity was detected (p = 0.02), indicating inconsistent effects in magnitude or direction. The I^2^ value of 62% suggests that most of the variability among studies is attributable to heterogeneity rather than random chance	⨁◯◯◯ Very low^b,d^	Important
Shoulder Pain and Disability Index (follow-up: mean six weeks; assessed with questionnaire) [2,8,9,13]									
4	RCT	Not serious	Not serious	Not serious	Not serious	None	A total of four studies were analyzed, comprising 87 subjects in the scapulothoracic cohort and 86 subjects in the comparison cohort. The standardized mean difference (SMD) was -0.87, with a 95% CI ranging from -1.55 to -0.19. The overall effect was statistically significant at p < 0.05. Substantial heterogeneity was observed, with 76% variability	⨁⨁⨁⨁ High	Important

Discussion

This review set out to answer a focused clinical question: Does adding scapulothoracic-targeted physical therapy to standard rehabilitation produce meaningful gains in pain, ROM, disability, and scapular kinematics in patients with AC? The corrected quantitative findings indicated a clear and consistent benefit for shoulder flexion ROM and functional disability, with the SPADI results supported by high-certainty evidence. Sensitivity analyses strengthen confidence in the abduction finding, whereas the evidence for pain and external rotation ROM remains uncertain.

Ferlito et al. [4] reported greater pain reduction with scapular PNF and mobilization than with conventional treatment. Our meta-analysis, however, found no statistically significant between-group differences in pain and had considerable heterogeneity. This discrepancy likely reflects the inclusion of studies with divergent pain outcomes, some favoring the intervention and others showing minimal difference. It may partly reflect methodological differences, particularly due to inclusion of studies assessing only immediate or acute effects [1], where sufficient time may not have been available for clinically meaningful pain modulation. The very low-GRADE certainty for this outcome cautions against drawing firm conclusions. Pain in AC is multifactorial and may be less responsive to biomechanical correction than to mobility and function.

The ROM findings align with the broader literature. Ferlito et al. [4] reported that eight of nine studies in their review showed significant improvement in at least one ROM plane (flexion, abduction, or external rotation). The one exception was Zaghloul et al. [7], in which MWM demonstrated superior outcomes compared with scapular mobilization. This pattern is consistent with a systematic review by Satpute et al. [16] who found not just statistical, but also clinically meaningful improvements in flexion and abduction ROM after MWM. The ROM improvements observed qualitatively in the present review may be explained by a reduction in scapulothoracic adhesions and by improvements in proprioceptive and kinesthetic feedback facilitating neuromuscular control [2,17,18,19]. Furthermore, scapular involvement is biomechanically greatest during flexion and abduction rather than external rotation, which may explain the more consistent gains observed in these planes [20]. External rotation remained consistently non-significant, possibly reflecting the dominant role of glenohumeral capsular tightness in limiting this movement plane, a component less directly addressed by scapulothoracic interventions.

The most compelling finding in this review is the significant, high-certainty SPADI result. This moderate-to-large effect represents a clinically meaningful reduction in shoulder pain and disability and is consistent across all four contributing studies. One limitation of our results was that they could not be directly compared with minimal clinically important difference (MCID) scores for any outcome measure because we standardized mean scores in our meta-analysis.

Five studies assessed scapular kinematics using heterogeneous methods [1,2,8,10,13]. Scapular upward rotation, as assessed by the LSST, did not differ significantly between groups in studies by Balcı et al. and Akbaş et al. [1,8]. The LSST reflects not only joint stiffness but also muscle control, pain, posture, and underlying shoulder mechanics. Additional confounders, including thoracic stiffness and muscle activation patterns, may modify the response to treatment. While LSST demonstrates acceptable reliability, its clinical utility as a diagnostic tool in this context remains limited [21]. For SHR, two studies [2,13] demonstrated improvement, with a difference of four degrees considered clinically significant. The addition of taping to dynamic scapular recognition exercises further improved SHR, consistent with the proposed mechanism whereby enhanced scapular upward rotation reduces the mechanical load on a stiff glenohumeral capsule by redistributing movement demand to the scapulothoracic articulation [22]. The study by Yang et al. [10] found improvements in posterior tipping and scapular upward rotation in the criteria intervention group, which received a combination of scapular mobilization and end-range mobilization.

Limitations

This review has several important limitations. The majority of the included studies had small sample sizes, limiting the meta-analysis' statistical power. High clinical and methodological heterogeneity precluded the synthesis of several key outcomes. Four of the nine studies were rated as high risk of bias, and six were not registered with a clinical trials registry, raising concerns about selective reporting, collectively reducing the certainty of the pooled estimates. Meta-analysis was conducted using NetMetaEasy, a network meta-analysis platform. Although the software produces results equivalent to conventional pairwise meta-analysis for two-arm comparisons, readers should note that this tool is not yet as widely validated for pairwise analyses as established platforms such as RevMan (The Cochrane Collaboration, London, England, UK) or R (R Foundation for Statistical Computing, Vienna, Austria, https://www.R-project.org/). Results were therefore cross-checked manually against the pooled SMD formula to confirm accuracy.

## Conclusions

This review provides meaningful evidence that physical therapy targeting the scapulothoracic joint offers a significant additive benefit over conventional rehabilitation for shoulder flexion ROM and functional disability, as measured by the SPADI, in patients with AC. The SPADI finding is supported by high-certainty GRADE evidence, making it the most clinically actionable result of this review. Sensitivity analyses further suggest that the abduction benefit may be underestimated in the primary analysis. Taken together, these results provide a clinically grounded rationale for routinely incorporating scapulothoracic interventions into rehabilitation programs for AC. Future high-quality RCTs with larger sample sizes, standardized protocols, prospective registration, and harmonized outcome assessment are needed to confirm the benefits of flexion and abduction, clarify whether scapulothoracic approaches meaningfully reduce pain or improve external rotation, and determine whether these benefits are sustained beyond the short term.
